# Divergent effects of glutathione depletion on isocitrate dehydrogenase 1 and the pentose phosphate pathway in hamster liver

**DOI:** 10.14814/phy2.14554

**Published:** 2020-08-18

**Authors:** Eunsook S. Jin, Min H. Lee, Craig R. Malloy

**Affiliations:** ^1^ Advanced Imaging Research Center University of Texas Southwestern Medical Center Dallas TX USA; ^2^ Department of Internal Medicine University of Texas Southwestern Medical Center Dallas TX USA; ^3^ Department of Radiology University of Texas Southwestern Medical Center Dallas TX USA; ^4^ VA North Texas Health Care System Dallas TX USA

**Keywords:** acetaminophen, antioxidant activity, glycerol, mitochondria, NADPH‐producing enzyme

## Abstract

The liver regenerates NADPH via multiple pathways to maintain redox balance and reductive biosynthesis. The pentose phosphate pathway (PPP) contributes to hepatic lipogenesis by supplying NADPH, and it is thought to play a major role in response to oxidative stress. This study determined the significance of the PPP and related NADPH‐regenerating enzymes in the liver under oxidative stress. Fasted hamsters received acetaminophen (400 mg/kg) to deplete glutathione in the liver and [U‐^13^C_3_]glycerol to measure the PPP activity by analysis of ^13^C distribution in plasma glucose. Blood and liver were harvested to assess NADPH‐producing enzymes, antioxidant defense, PPP, and other relevant biochemical processes. Acetaminophen caused glutathione depletion and decreased activities of glutathione peroxidase and catalase in the liver, but it did not change triglyceride synthesis. Although the PPP is potentially an abundant source of NADPH, its activity was decreased and the expression of glucose 6‐phosphate dehydrogenase remained unchanged after acetaminophen treatment. The effects of acetaminophen on other NADPH‐producing enzymes were complex. Isocitrate dehydrogenase 1 was overexpressed, both isocitrate dehydrogenase 2 and malic enzyme 1 were underexpressed, and methylenetetrahydrofolate dehydrogenase 1 remained unchanged. In summary, isocitrate dehydrogenase 1 was most sensitive to glutathione depletion caused by acetaminophen, but glucose 6‐phosphate dehydrogenase, the regulatory enzyme of PPP, was not.

## Graphical abstract

Glutathione depletion in hamster liver led to divergent effects on proteins of NADPH‐producing enzymes; increased isocitrate dehydrogenase 1, decreased isocitrate dehydrogenase 2 and malic enzyme 1, and unchanged glucose 6‐phosphate dehydrogenase and methylenetetrahydrofolate dehydrogenase 1. Isocitrate dehydrogenase 1 was most sensitive to glutathione depletion caused by acetaminophen, but glucose 6‐phosphate dehydrogenase, the regulatory enzyme of PPP, was not.

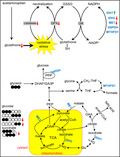

## 1 INTRODUCTION

The pentose phosphate pathway (PPP), a shunt of glycolysis, produces NADPH and pentoses. The PPP is potentially an abundant source of NADPH because metabolism of one glucose molecule through the pathway generates two NADPH. This reducing agent is regenerated from NADP^+^ at the levels of glucose 6‐phosphate dehydrogenase (G6PDH) and 6‐phosphogluconate dehydrogenase in the oxidative phase of the pathway. NADPH is essential for reductive biosynthesis and redox homeostasis. The PPP in erythrocytes is the sole source of NADPH for protection against oxidative stress and glutathione regeneration (Rogers et al., 2009), and the pathway is widely cited to play a pivotal role for antioxidant defense (Filosa et al., 2003; Grant, 2008). However, NADPH is also produced from other sources in many organs including malic enzyme (ME), isocitrate dehydrogenase (IDH), and methylenetetrahydrofolate dehydrogenase (MTHFD). In the presence of multiple alternative sources of NADPH, the significance of the PPP and these enzymes specifically for antioxidant defense is of great interest.

In addition to protection against oxidative stress, multiple hepatic processes require NADPH such as cholesterol synthesis, fatty acid elongation and detoxification. Earlier studies reported that the PPP and ME1 are essential for hepatic lipogenesis (Al‐Dwairi et al., 2014; Jin, Lee, Murphy, & Malloy, 2018; Kazumi et al., 1997). However, the sources of NADPH for antioxidant activity and glutathione regeneration in liver are less clear. This gap is worth investigation because oxidative stress or toxicity associated with drug overdose may induce severe liver disease. A simple method to induce oxidative stress in liver is the use of acetaminophen (acetyl‐p‐aminophenol; APAP). In therapeutic doses, acetaminophen is mainly glucuronidated or sulfated, which yields nontoxic metabolites excreted in the urine. The remaining small fraction of acetaminophen is oxidized to highly reactive *N*‐acetyl‐p‐benzoquinone imine that is detoxified through conjugation with glutathione. However, an overdose of acetaminophen saturates glucuronidation and sulfation pathways, depletes glutathione, and leads to oxidative damage (Davis, Potter, Jollow, & Mitchell, 1974; James, Mayeux, & Hinson, 2003; Mitchell, Jollow, Potter, Gillette, & Brodie, 1973), and it is the leading cause of acute liver failure (Lee, 2017).

Glutathione depletion after an acetaminophen overdose would be expected to induce PPP activity in the liver to counteract oxidative stress, but this hypothesis has not been investigated. The PPP activity is typically measured using a ^13^C‐labeled glucose tracer such as [1,2‐^13^C_2_]glucose and the labeling pattern in lactate is analyzed (Lee, Malloy, Corbin, Li, & Jin, 2019; Lee et al., 1998; Marin‐Valencia et al., 2012). Recently we introduced [U‐^13^C_3_]glycerol, a gluconeogenic substrate, for the assessment of hepatic PPP in experimental animals and human subjects by analyzing ^13^C‐labeling patterns in plasma glucose (Carreau et al., 2019; Jin, Sherry, & Malloy, 2014; Neeland, Hughes, Ayers, Malloy, & Jin, 2017). Glycerol in blood is utilized by the liver with highly expressed glycerol kinase. During gluconeogenesis from [U‐^13^C_3_]glycerol in the liver, a shunt through the PPP produces [1,2‐^13^C_2_]glucose from [1,2,3‐^13^C_3_]hexose reflecting the PPP activity (Jin et al., 2014). Because glucose synthesized in the liver is released into the circulation, the pattern of ^13^C‐labeling in plasma glucose is sensitive to hepatic PPP activity after [U‐^13^C_3_]glycerol administration. The labeling pattern in plasma glucose is also sensitive to other biochemical processes in the liver such as gluconeogenesis and metabolism through the TCA cycle (Jin et al., 2014).

The current study was designed to test the hypothesis that acetaminophen‐induced glutathione depletion increases PPP activity and NADPH‐producing enzymes in the hamster liver. The hamster was chosen for study because, like humans, the species is sensitive to an acetaminophen overdose (Madhu & Klaassen, 1991). All animals received [U‐^13^C_3_]glycerol for the assessments of multiple biochemical processes in the liver including the PPP. Acetaminophen depleted glutathione in the liver, but it had complex effects on NADPH‐producing enzymes and, unexpectedly, PPP activity was not increased.

## 2 MATERIALS AND METHODS

### 2.1 Animal studies

The study was approved by the Institutional Animal Care and Use Committee at the University of Texas Southwestern Medical Center. Male golden Syrian hamsters (130–160 g, *n* = 26) were purchased from Charles Rivers Laboratories (Wilmington, MA). All animals were placed on a 12:12‐hr day–night cycle and had free access to rodent chow and water. The day before the study day, chow was withdrawn at 4 p.m. and hamsters were fasted overnight with access to water. At 9 a.m. on the study day, a group of hamsters (*n* = 16) received acetaminophen (400 mg/kg) dissolved in warm saline (2ml) intraperitoneally under isoflurane anesthesia while the other group (*n* = 10) received saline only. Hamsters awakened quickly and were physically active. After waiting for 2 hr, all hamsters received [U‐^13^C_3_]glycerol (50% or 99%, 100 mg/kg; Cambridge Isotopes, Andover, MA) dissolved in deionized water (1 ml) intraperitoneally under anesthesia. They again awakened quickly and were ambulatory for an hour prior to sacrifice under anesthesia. Whole blood was harvested from the inferior vena cava, and liver was excised and freeze‐clamped using liquid nitrogen. Plasma and liver tissue were kept at −80°C prior to sample preparation.

### 2.2 Sample processing for NMR analysis

Ground liver tissue (3 g) was treated with cold perchloric acid (10%, 15 ml) to extract water‐soluble metabolites. Plasma (4–5 ml) was also treated with perchloric acid (70%) to a final concentration of 10%. The mixture was vortexed for 1 min, centrifuged, and the supernatant was transferred to a new tube. The extraction was repeated, and the combined supernatant was neutralized using KOH solutions. After centrifuging to precipitate salts, supernatant was transferred and lyophilized for nuclear magnetic resonance (NMR) acquisition. The samples from plasma were further processed to convert glucose to monoacetone glucose as reported previously (Jin, Burgess, Merritt, Sherry, & Malloy, 2005). Briefly, plasma glucose was purified by passage through a column made of cation‐exchange resin (2 ml; Dowex 50Wx8‐200; Sigma) and anion‐exchange resin (2 ml; Amberlite IRA‐67; Sigma) using deionized water (120 ml) as eluent, and the effluent was lyophilized. Dried glucose was suspended in acetone (3 ml) containing concentrated sulfuric acid (120 µl), and the mixture was stirred for 4 hr at room temperature. After adding deionized water (3 ml) to the mixture, Na_2_CO_3_ (1.5 M) was added to increase pH up to 2.0. The mixture was stirred for 24 hr to produce monoacetone glucose followed by increasing pH up to 8.0 with Na_2_CO_3_. Dried monoacetone glucose was extracted using hot ethyl acetate (5x) and purified by passage through a 3‐ml DSC‐18 cartridge using acetonitrile (5%) as eluent, and the effluent was dried.

Ground liver tissue (2 g) was treated with a mixture of chloroform and methanol (2:1; 8 ml) to extract lipids, and stirred for at least 30 min. The mixture was centrifuged at a low speed, and chloroform layer was transferred to a new glass vial. The extraction was repeated after adding chloroform (5 ml), and the combined extracts were dried using vacuum with a liquid nitrogen trap.

### 2.3 NMR spectroscopy

NMR spectra were collected using a 14.1T spectrometer (Varian INOVA, Agilent, Santa Clara, CA) equipped with a 3‐mm broadband probe with the observe coil tuned to ^13^C (150 MHz). Samples of perchloric acid extracts were dissolved in ^2^H_2_O (300 μL for liver extracts; 200 μL for plasma extracts) containing 4,4‐dimethyl‐4‐silapentane‐1‐sulfonic acid (DSS; 5 mM) as a NMR reference, centrifuged and supernatant was transferred to a 3‐mm tube for NMR acquisition. ^13^C NMR spectra were acquired using a 45° pulse, a 36,765‐Hz sweep width, 55,147 data points, and a 1.5‐sec acquisition time with 1.5‐s interpulse delay at 25°C. Proton decoupling was performed using a standard WALTZ‐16 pulse sequence. Monoacetone glucose dissolved in a mixture of deuterated acetonitrile (150 µl) and deionized water (10 µl) was transferred to a NMR tube. ^13^C NMR of monoacetone glucose was acquired with a 52° pulse, a 20,330‐Hz sweep width, 60,992 data points, and a 1.5‐s acquisition time with 1.5‐s interpulse delay at 25°C. Lipid extracts were dissolved in deuterated chloroform (CDCl_3_, 170 µl), and ^13^C NMR spectra of lipids were acquired using a 60° pulse, a 36,765‐Hz sweep width, 110,294 data points and a 1.5‐s acquisition time with 1.5‐s interpulse delay at 25°C. Spectra were averaged at least 8,000 scans requiring 7 hr. All NMR spectra were analyzed using ACD/Labs NMR spectral analysis program (Advanced Chemistry Development, Inc., Toronto, Canada).

### 2.4 NMR assessments for hepatic PPP, gluconeogenesis, glutathione, and other metabolites

**Figure 1:**
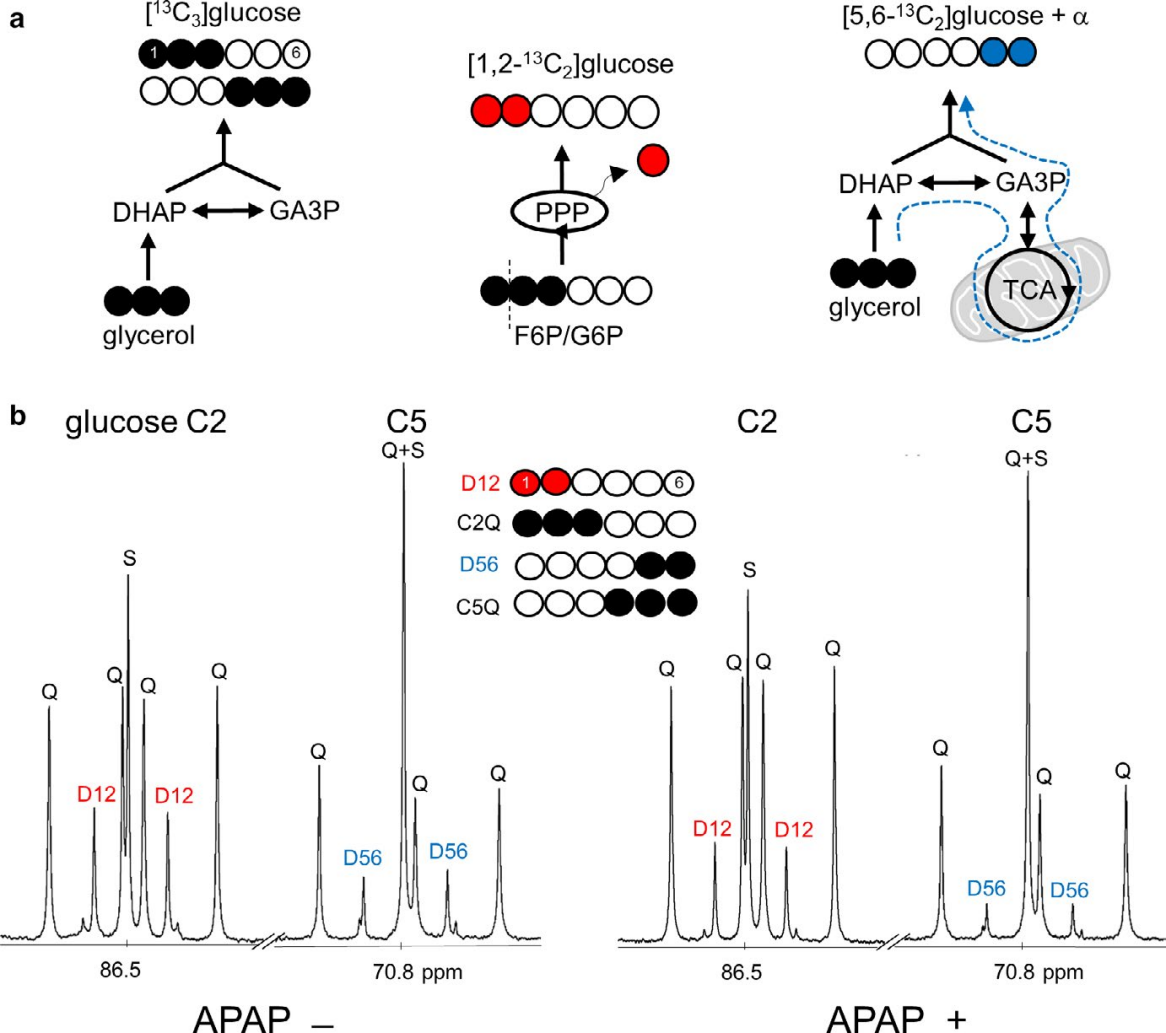
[U‐^13^C_3_]glycerol incorporation to glucose informing biochemical processes in the liver. (a) [U‐^13^C_3_]glycerol direct incorporation to gluconeogenesis produces triple‐labeled ([1,2,3‐^13^C_3_] and [4,5,6‐^13^C_3_]) glucose. The entry of [1,2,3‐^13^C_3_]glucose 6‐phosphate (G6P) to the PPP yields [1,2‐^13^C_2_]glucose. The glycerol metabolism through the TCA cycle prior to gluconeogenesis produces double‐labeled glucose isotopomers including [5,6‐^13^C_2_]glucose. (b) ^13^C NMR of glucose derivative from a control and an acetaminophen‐treated hamster show signals from ^13^C‐glucose after the administration of [U‐^13^C_3_]glycerol. Both the PPP and metabolism through the TCA cycle produce [1,2‐^13^C_2_]glucose, but [1,2‐^13^C_2_]glucose produced through the PPP can be distinguished because the PPP activity increases [1,2‐^13^C_2_]glucose relative to [5,6‐^13^C_2_]glucose. Abbreviations: D12, [1,2‐^13^C_2_]glucose; D56, [5,6‐^13^C_2_]glucose; Q, [1,2,3‐^13^C_3_]glucose at C2 or [4,5,6‐^13^C_3_]glucose at C5; S, [2‐^13^C_1_]glucose at C2 or [5‐^13^C_1_]glucose at C5; APAP, acetyl‐p‐aminophenol (acetaminophen); DHAP, dihydroxyacetone phosphate; F6P, fructose 6‐phosphate; GA3P, glyceraldehyde 3‐phosphate; open circle, ^12^C; black circle, ^13^C; red circle, ^13^C after experiencing the PPP; blue circle, ^13^C after experiencing the TCA cycle

Hepatic PPP activity and gluconeogenesis from glycerol were measured using ^13^C NMR analysis of monoacetone glucose derived from plasma glucose as reported previously (Jin et al., 2014). Briefly, [U‐^13^C_3_]glycerol direct incorporation into gluconeogenesis produces triple‐labeled ([1,2,3‐^13^C_3_] and [4,5,6‐^13^C_3_]) glucose (Figure 1a). A fraction of [U‐^13^C_3_]glycerol is metabolized through the tricarboxylic acid (TCA) cycle prior to gluconeogenesis, which produces double‐labeled glucose including [5,6‐^13^C_2_]glucose and [1,2‐^13^C_2_]glucose. Among these glucose isotopomers, [5,6‐^13^C_2_]glucose reflects biosynthetic function through the TCA cycle in mitochondria, but [1,2‐^13^C_2_]glucose does not because it is also produced through the PPP. The entry of [1,2,3‐^13^C_3_]glucose 6‐phosphate into the PPP yields [1,2‐^13^C_2_]fructose 6‐phosphate and consequently [1,2‐^13^C_2_]glucose. This fraction of [1,2‐^13^C_2_]glucose produced through the PPP ([1,2‐^13^C_2_]glucose_ppp_) can be distinguished from that produced through the TCA cycle because the activity of PPP increases the fraction of [1,2‐^13^C_2_]glucose relative to [5,6‐^13^C_2_]glucose (Jin et al., 2014). ^13^C NMR analysis of monoacetone glucose allows the quantitation of all these glucose isotopomers (Figure 1b).

**Figure 2:**
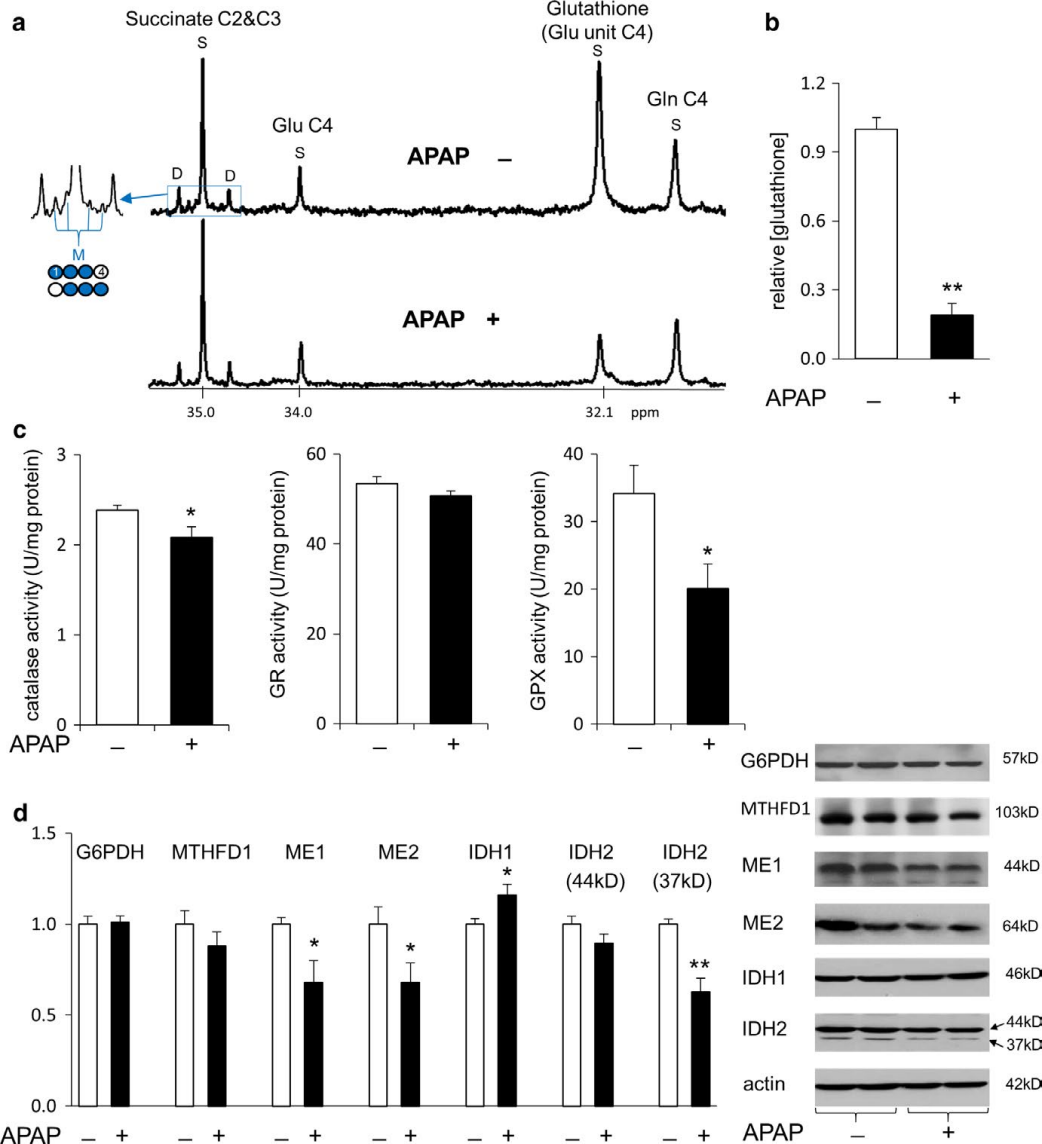
Suppressed antioxidant activity in the liver with acetaminophen treatment. (a) ^13^C NMR spectra of liver extracts show singlet (S) signals from glutathione, succinate, and glutamate (Glu) with natural ^13^C abundance. Multiplets including the signal from [1,2,3‐^13^C_3_]‐ and [2,3,4‐^13^C_3_]succinate (M) are also detected at succinate C2 and C3 region. (b) Acetaminophen treatment almost depleted glutathione in the liver. (c) Acetaminophen decreased the activities of catalase and glutathione peroxidase (GPX), but not the activity of glutathione reductase (GR). (d) The protein expression of IDH1 was increased with acetaminophen treatment while proteins of ME1/2 and IDH2 were reduced. Both G6PDH and MTHFD1 remained unchanged. Two bands from IDH2 are from precursor (44 kD) and a mature form (37 KD). Abbreviations: IDH1, cytosolic NADP^+^‐dependent isocitrate dehydrogenase; IDH2, mitochondrial NADP^+^‐dependent isocitrate dehydrogenase; ME1, cytosolic NADP^+^‐dependent malic enzyme; ME2, mitochondrial NAD^+^‐dependent malic enzyme; MTHFD1, methylenetetrahydrofolate dehydrogenase 1; Gln, glutamine; *, *p* < .05, **, *p* < .001; *n* = 6–7 in each group

The relative concentrations of water‐soluble metabolites in the liver were measured using NMR analysis of tissue extracts (Figure 2a). In ^13^C NMR spectra, a singlet (S) from each metabolite was normalized by DSS to measure the pool size of glutathione, succinate, or glutamate. In the assessment of glutathione, signal from glutamate unit (32.1 ppm) was quantified and total glutathione (GSH and GSSG) was reported in this study because of expected oxidation during sample preparation. ^13^C enrichment in succinate was calculated using multiplet (M in Figure 2a, signal from [1,2,3‐^13^C_3_]‐ and [2,3,4‐^13^C_3_]succinate) by assuming its singlet as natural abundance (1.1%) since the probability of producing single‐labeled molecule from exogenous [U‐^13^C_3_]glycerol was negligible.

### 2.5 NMR analysis of lipid extracts from the liver

**Figure 3:**
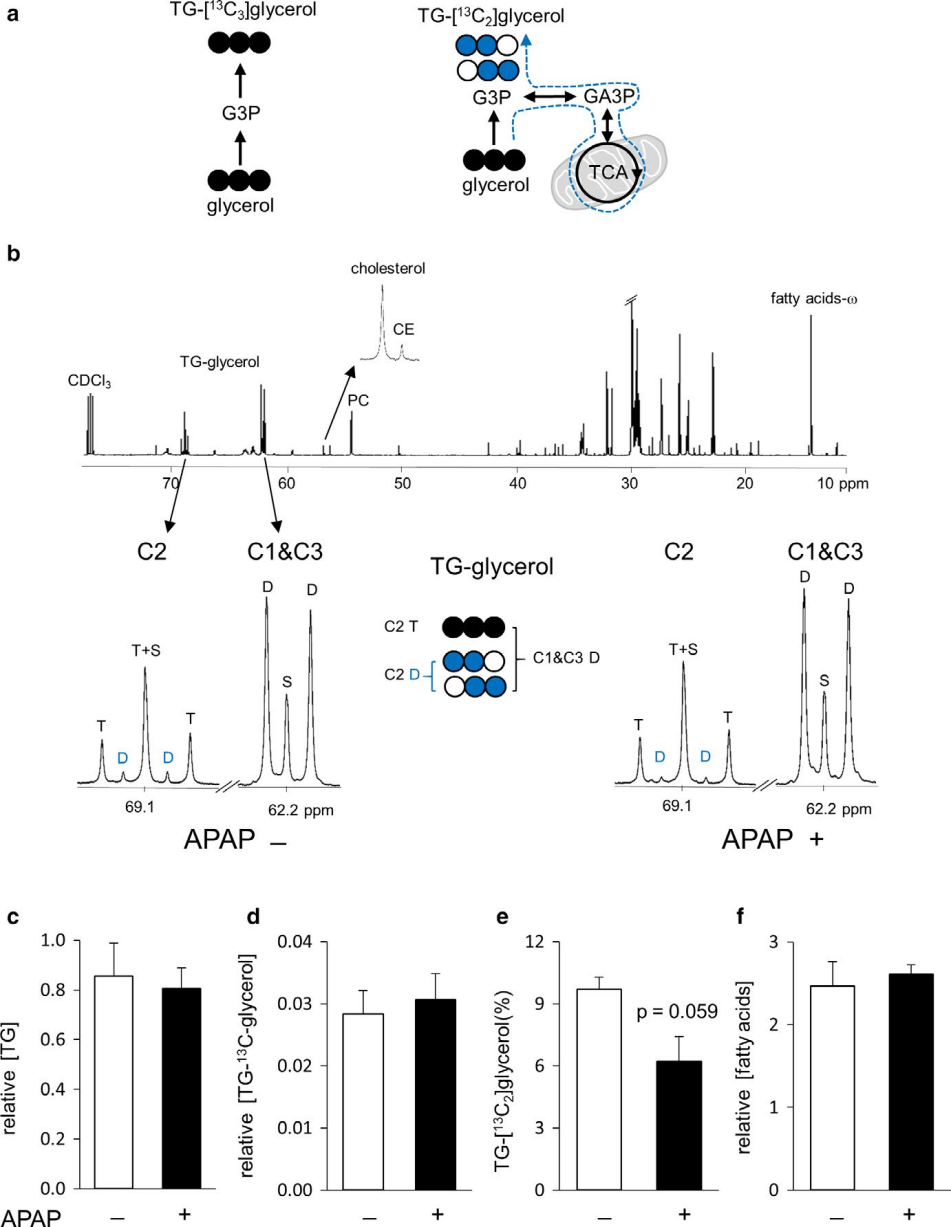
NMR analysis of lipid extracts from the liver. (a) [U‐^13^C_3_]glycerol direct incorporation to triglycerides (TG) produces triple‐labeled ([U‐^13^C_3_]) glycerol backbones. [U‐^13^C_3_]glycerol metabolism through the TCA cycle prior to TG produces double‐labeled backbones. (b) ^13^C NMR of lipid extracts shows signals from the glycerol backbones of triglycerides (TG‐glycerol), cholesterol, cholesterol ester (CE), phosphatidylcholine (PC), and fatty acids. Multiplets at TG‐glycerol demonstrate [U‐^13^C_3_]glycerol incorporation to TG. (c and d) The levels of TG and TG‐[^13^C]glycerol in the liver were similar between controls and acetaminophen‐treated animals. (e) The fraction of TG‐[^13^C_2_]glycerol tended to be reduced in acetaminophen‐treated animals without a statistical significance. The double‐labeled backbones of TG were produced through [U‐^13^C_3_]glycerol metabolism in the TCA cycle. (f) The concentrations of fatty acids remained unchanged with acetaminophen treatment. Abbreviations: D at C1 and C3, [U‐^13^C_3_]‐, [1,2‐^13^C_2_]‐, and [2,3‐^13^C_2_]glycerol; D at C2, [1,2‐^13^C_2_]‐ and [2,3‐^13^C_2_]glycerol; S, [2‐^13^C_1_]glycerol at C2 or [1‐^13^C_1_]‐ and [3‐^13^C_1_]glycerol at C1 and C3; T at C2, [U‐^13^C_3_]glycerol; G3P, glycerol 3‐phosphate; open circle, ^12^C; black circle, ^13^C; blue circle, ^13^C after experiencing the TCA cycle; *n* = 3 in each group

[U‐^13^C_3_]glycerol incorporation to triglycerides (TG) was examined using NMR analysis of the glycerol backbones of triglycerides (TG‐glycerol) from the liver. The glycerol direct incorporation to TG produces triple‐labeled backbones, TG‐[^13^C_3_]glycerol, while indirect contribution through the TCA cycle leads to double‐labeled backbones, TG‐[^13^C_2_]glycerol (Figure 3a). The level of TG was calculated using singlet at TG‐glycerol C1 and C3 normalized by solvent signal (CDCl_3_/3) at 77.2 ppm. TG containing ^13^C‐labeled glycerol backbones (TG‐[^13^C]glycerol) was measured using doublet at C1 and C3 assuming the singlet as natural ^13^C abundance. The percentage of TG‐[^13^C_2_]glycerol, indirect contribution through the TCA cycle, was calculated using the ratio of doublet/(doublet + triplet) at the glycerol backbone C2 whereas doublet (D) is TG‐[^13^C_2_]glycerol and triplet (T) is TG‐[^13^C_3_]glycerol (Figure 3b). The levels of other lipids of interests were measured using signals from fatty acid ω carbon (‐CH_3_, 14.2 ppm), phosphatidylcholine ‐N^+^(CH_3_)_3_ (54.4 ppm), cholesterol ester C14 (56.8 ppm), and cholesterol C14 (56.9 ppm) normalized by solvent signal (CDCl_3_/3) in ^13^C NMR spectra (Figure 3b).

### 2.6 Immunoblot assay

Liver tissue was prepared using RIPA buffer supplemented with a protease inhibitor cocktail and a phosphatase inhibitor cocktail (Roche Diagnostics, Indianapolis, IN). Proteins were mixed with sodium dodecyl sulfate buffer and denatured by boiling for 5 min. Proteins in the sample were separated by 8%–15% SDS‐PAGE gels and transferred onto a nitrocellulose membrane (Millipore, Billerica, MA). Membranes were blocked in 5% nonfat milk in Tris‐buffered saline with 0.1% Tween 20 (TBS/T) for 1 hr. After blocking, blots were incubated with primary antibodies in diluted blocking buffer overnight at 4°C, washed in TBS/T, and incubated with secondary antibodies conjugated to horseradish peroxidase (HRP) for 1 hr at room temperature. Blots were developed using an Immobilon Western Chemiluminescent HRP substrate (Millipore). Relative densities of bands were quantified with UN‐SCAN‐IT Gel 6.1 software, and normalized by actin intensity of the same sample. The following antibodies were used: IDH2 (Abcam), G6PDH, IDH1 (Cell Signaling), ME1 (Santa Cruz Biotechnology), MTHFD1 (Abgent), ME2, actin, rabbit IgG HRP‐linked whole antibody, and mouse IgG HRP‐linked whole antibody (Sigma).

### 2.7 Enzyme activity measurement and other assays

The activities of catalase, glutathione peroxidase, and glutathione reductase were assayed using commercial kits (Cayman Chemical, Ann Arbor, MI) with Synergy H1 Hybrid reader at absorbance at 340 nm or 540 nm. Plasma glucose was measured using glucose oxidase method (YSI 2300 Glucose Analyzer; GMI, Inc), and plasma insulin was measured using a rodent insulin ELISA kit (ALPCO). Aspartate aminotransferase (AST) and alanine aminotransferase (ALT) were measured using the Vitros 250 analyzers (Johnson & Johnson).

### 2.8 Statistics

Normal distribution of data was verified with the values of skewness and kurtosis between −2 and + 2. Data are expressed as mean ± SE. Comparisons between two groups were made using a student's *t* test, where *p* < .05 was considered significant.

## 3 RESULTS

### 3.1 Divergent effects of glutathione depletion on NADPH‐producing enzymes in livers

**Figure 4:**
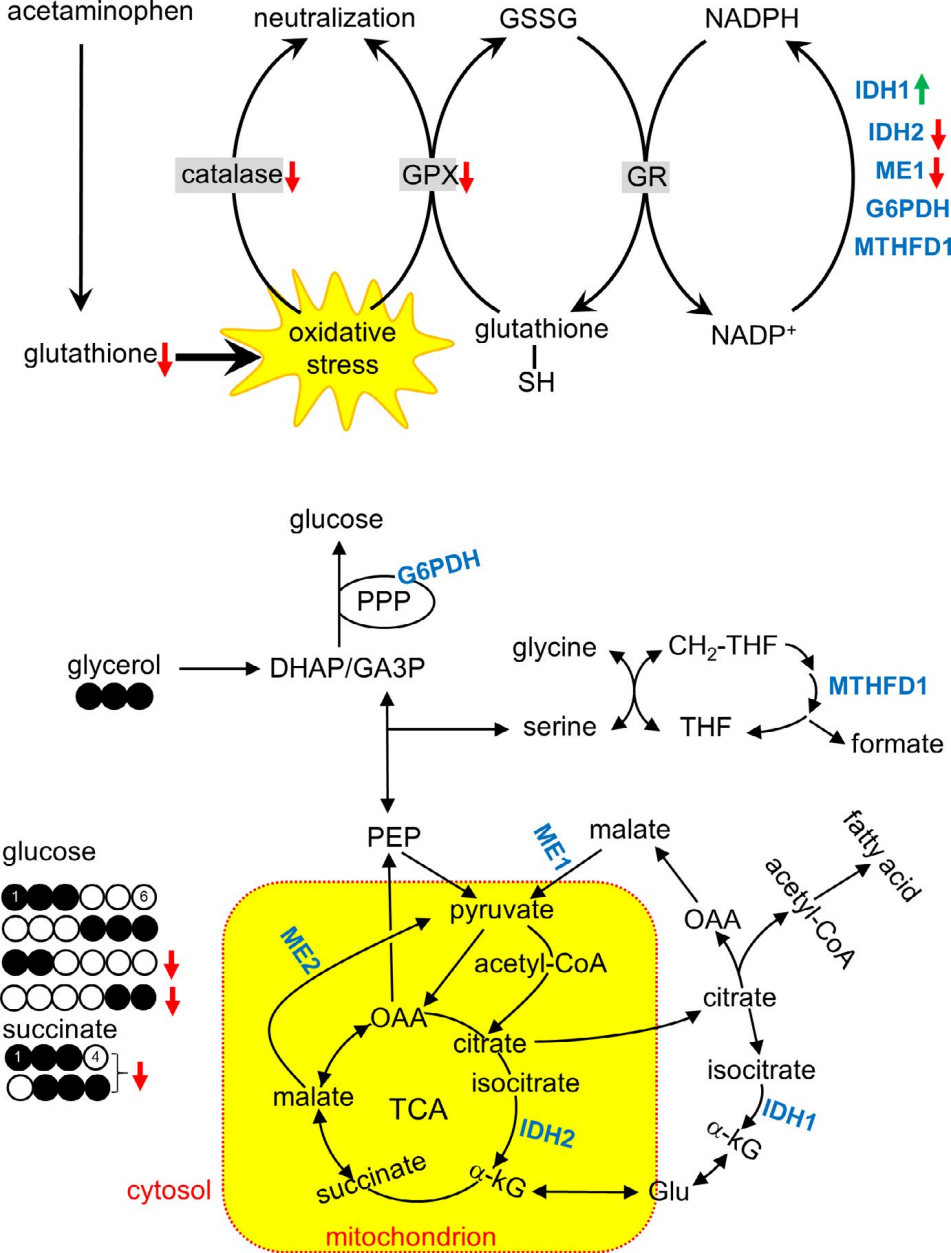
Illustration of the effects of acetaminophen on glutathione, antioxidant processes, and metabolic processes. Acetaminophen depleted glutathione in the liver causing oxidative stress. It also reduced the activities of catalase and glutathione peroxidase (GPX) that neutralize oxygen radicals, but did not change the activity of glutathione reductase (GR) that requires NADPH to regenerate reduced glutathione. Responses of NADPH‐producing enzymes were complex; increased isocitrate dehydrogenase 1 (IDH1), decreased IDH2 and malic enzyme 1 (ME1), and unchanged glucose 6‐phosphate dehydrogenase (G6PDH) and methylenetetrahydrofolate dehydrogenase 1 (MTHFD1). According to NMR analysis of glucose, the production of triple‐labeled ([1,2,3‐^13^C_3_] and [4,5,6‐^13^C_3_]) glucose remained unchanged with acetaminophen treatment, informing intact gluconeogenesis directly from [U‐^13^C_3_]glycerol in the cytosol. However, both [1,2‐^13^C_2_]glucose produced through the PPP and [5,6‐^13^C_2_]glucose produced through the TCA cycle were suppressed. In NMR analysis of liver extracts, triple‐labeled ([1,2,3‐^13^C_3_] and [2,3,4‐^13^C_3_]) succinate was reduced with acetaminophen, informing suppressed pyruvate carboxylation to enter the TCA cycle. Abbreviation: DHAP, dihydroxyacetone phosphate; GA3P, glyceraldehyde 3‐phosphate; Glu, glutamate; GSSG, glutathione disulfide; α‐kG, alpha‐ketoglutarate; OAA, oxaloacetate; PEP, phosphoenolpyruvate; THF, tetrahydrofolate

Acetaminophen treatment caused multiple changes in antioxidant processes and biochemical reactions in the liver of hamsters (Figure 4). Acetaminophen almost completely depleted glutathione (Figure 2b). It also reduced the activities of catalase and glutathione peroxidase, but the activity of glutathione reductase remained unchanged (Figure 2c). The protein expression of cytosolic NADP^+^‐dependent IDH1 was increased by acetaminophen treatment while cytosolic NADP^+^‐dependent ME1, mitochondrial NAD^+^‐dependent ME2, and mitochondrial NADP^+^‐dependent IDH2 were decreased. However, proteins of G6PDH and MTHFD1 remained unchanged by acetaminophen (Figure 2d). The levels of ALT, AST, and insulin in plasma were not significantly changed by acetaminophen treatment (Table 1).

**Table 1:** Biochemical analysis of plasma

	APAP ˗ (*n* = 5–8)	APAP + (*n* = 6–10)	*p*
ALT (U/L)	84 ± 9	90 ± 12	.709
AST (U/L)	62 ± 8	54 ± 5	.378
Insulin (ng/mL)	0.3 ± 0.1	0.9 ± 0.3	.077

Note

Values are means ± SE.

Abbreviations: ALT, alanine aminotransferase; APAP, acetyl‐p‐aminophenol (acetaminophen); AST, aspartate aminotransferase.

### 3.2 Reduced PPP activity and metabolism through the TCA cycle in glutathione‐depleted livers

**Figure 5:**
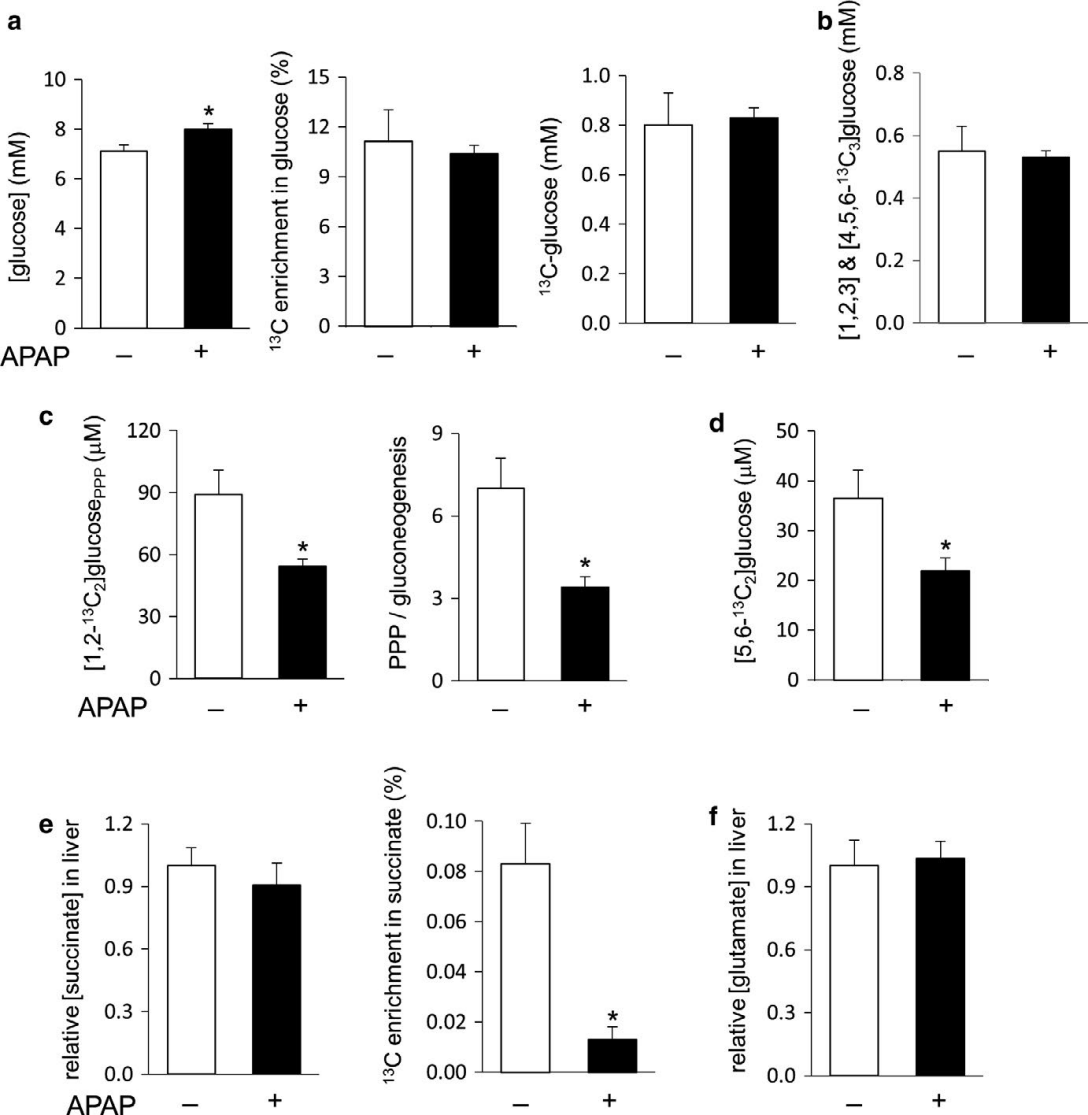
Intact gluconeogenesis, but reduced PPP and the TCA cycle activities in the liver with acetaminophen treatment. (a) Plasma glucose was slightly increased with acetaminophen treatment, but ^13^C‐glucose derived from [U‐^13^C_3_]glycerol was unchanged. (b) The sum of [1,2,3‐^13^C_3_] and [4,5,6‐^13^C_3_]glucose remained unaltered with acetaminophen treatment. (c) Acetaminophen reduced [1,2‐^13^C_2_]glucose production through the PPP and the ratio of PPP/gluconeogenesis from glycerol. (d) Acetaminophen decreased [5,6‐^13^C_2_]glucose production through the TCA cycle. (e) The concentration of succinate in the liver remained unchanged after acetaminophen treatment, but ^13^C enrichment by triple‐labeled succinate was reduced. (f) The concentration of glutamate in the liver remained unchanged with acetaminophen treatment. *p < .05; *n* = 5–10 in each group

Hamsters with acetaminophen treatment had slightly higher plasma glucose concentration than controls, but ^13^C enrichment in glucose and the concentration of ^13^C‐labeled glucose remained unchanged with acetaminophen treatment (Figure 5a). The sum of [1,2,3‐^13^C_3_]‐ and [4,5,6‐^13^C_3_]glucose in the acetaminophen group was also similar with controls (Figure 5b), demonstrating intact gluconeogenesis directly from glycerol. However, hamsters with acetaminophen had lower [1,2‐^13^C_2_]glucose production through the PPP and lower ratio of PPP/gluconeogenesis from glycerol than controls (Figure 5c). [5,6‐^13^C_2_]glucose production through the TCA cycle was also decreased in animals with acetaminophen (Figure 5d). A subset of hamsters (two controls and six acetaminophen‐treated animals) was excluded from these measurements because ^13^C enrichment in glucose was not sufficient for reliable analysis.

The TCA cycle intermediates and metabolites in exchange with the cycle were also examined. The entry of [U‐^13^C_3_]pyruvate to the TCA cycle through carboxylation produces triple‐labeled four carbon units including succinate (Figure 2a). Alternatively, the entry through decarboxylation produces [1,2‐^13^C_2_]acetyl‐CoA and consequently [4,5‐^13^C_2_]α‐ketoglutarate through the TCA cycle. Since α‐ketoglutarate is in exchange with glutamate, the detection of [4,5‐^13^C_2_]glutamate is evidence of pyruvate decarboxylation with the use of [U‐^13^C_3_]glycerol. Signal from triple‐labeled succinate was weak (Figure 2a), but sufficient to indicate active pyruvate carboxylase in the liver. The level of succinate remained unchanged with acetaminophen treatment, but ^13^C enrichment by triple‐labeled ([1,2,3‐^13^C_3_] and [2,3,4‐^13^C_3_]) succinate was reduced (Figure 5e). The level of glutamate also remained unchanged with acetaminophen treatment (Figure 5f), and [4,5‐^13^C_2_]glutamate was not detected in the liver of either controls or acetaminophen‐treated animals.

### 3.3 Minimal changes in lipid metabolism in glutathione‐depleted livers

TG synthesis in the liver was studied using ^13^C NMR analysis of the glycerol backbones of TG (Figure 3b). The level of TG remained unchanged after acetaminophen treatment (Figure 3c), and the level of newly synthesized TG containing [^13^C]glycerol was also unchanged (Figure 3d). However, among the newly synthesized TG, the percentage of [U‐^13^C_3_]glycerol incorporation to TG indirectly through the TCA cycle tended to be lower in acetaminophen‐treated livers without a statistical significance (Figure 3e). There were no differences in the levels of other lipids of interest including phosphatidylcholine, cholesterol ester, cholesterol, and fatty acids (Figure 3f).

## 4 DISCUSSION

This study tested the popular hypothesis that the PPP plays a significant role in hepatic protection under oxidative stress using hamsters receiving an acetaminophen overdose and metabolizing [U‐^13^C_3_]glycerol. Acetaminophen reduced the concentration of glutathione, as expected, and the activities of catalase and glutathione peroxidase in the liver were also decreased. Glutathione depletion was associated with disruption of NADPH‐producing enzymes, but the effects on protein expressions of specific enzymes were complex. IDH1 was increased, both ME1 and IDH2 were decreased, and G6PDH and MTHFD1 remained unchanged. Hepatic PPP activity was reduced with acetaminophen treatment despite unchanged G6PDH. Together these data demonstrated that glutathione depletion in the liver led to divergent effects on NADPH‐regenerating enzymes and it did not induce the PPP activity.

NMR analysis of plasma glucose and liver metabolites revealed distinct effects of acetaminophen on biochemical processes in the cytosol and the mitochondrion. Gluconeogenesis directly from [U‐^13^C_3_]glycerol occurs in the cytosol, which was intact in the acetaminophen group based on [1,2,3‐^13^C_3_]‐ and [4,5,6‐^13^C_3_]glucose production (Figure 5b). Fatty acid esterification in the cytosol was also intact with unchanged production of TG‐[^13^C]glycerol (Figure 3d). [U‐^13^C_3_]pyruvate from the glycerol entered the TCA cycle via carboxylation based on the presence of triple‐labeled succinate and the lack of [4,5‐^13^C_2_]glutamate in the liver (Figure 2a). However, reduced [5,6‐^13^C_2_]glucose and the fraction of TG‐[^13^C_2_]glycerol produced through the TCA cycle with acetaminophen treatment (Figures 5d and 3e) demonstrated impaired biosynthetic functions in mitochondria. Consistent with these findings, triple‐labeled succinate of the TCA cycle was also reduced in the liver with acetaminophen treatment (Figure 5e). As biochemical processes in the cytosol and the mitochondrion responded distinctly to acetaminophen, so did the protein expressions of enzymes. The two isoforms of NADP^+^‐dependent IDH catalyze the conversion of isocitrate to α‐ketoglutarate in the cytosol by IDH1 and in the mitochondrion by IDH2 (Figure 4). Intriguingly IDH1 was overexpressed while IDH2 underexpressed after acetaminophen treatment. These distinct responses of IDH isoforms could be related to different levels of oxidative stress in their locations of the cell because mitochondria are particularly susceptible to reactive oxidative species with an acetaminophen overdose (Burcham & Harman, 1990; Du, Ramachandran, & Jaeschke, 2016; Hinson, Roberts, & James, 2010; Yoon, Babar, Choudhary, Kutner, & Pyrsopoulos, 2016). When oxidative stress exceeds the capacity of antioxidant system in mitochondria, it attacks and degrades proteins causing impaired biochemical processes and functions (Guo, Sun, Chen, & Zhang, 2013; Venditti, Di Stefano, & Di Meo, 2013). Cytosolic IDH1 was reported to play a role in antioxidant defense in cell studies (Lee et al., 2002) and to be much more active in NADPH production than G6PDH and ME (Veech, Eggleston, & Krebs, 1969). Overexpressed IDH1 in this study confirmed its role for protection against oxidative stress. Mitochondrial IDH2 was also reported to play a role in redox balance and the deficiency of this enzyme exacerbated acetaminophen hepatotoxicity (Jo et al., 2001; Kim, Lee, & Park, 2019). However, the underexpressed IDH2 in this study is consistent with the reported protein degradation by excessive oxygen radicals in mitochondria. Somewhat consistent with different expressions of these IDH isoforms, mitochondrial ME2 was underexpressed while cytosolic G6PDH and MTHFD1 remained unchanged after acetaminophen treatment.

ME1, however, was reduced with acetaminophen treatment, unlike other cytosolic enzymes. The role of ME1 in hepatic lipogenesis is known (Al‐Dwairi et al., 2014; Jin et al., 2018; Kazumi et al., 1997) and its dissociation from acetaminophen‐induced oxidative stress was also reported previously (Qian et al., 2008). In this study, acetaminophen treatment did not change lipid contents and fatty acid esterification using [U‐^13^C_3_]glycerol (Figure 3). However, earlier studies reported that an acetaminophen overdose disrupted lipid metabolism including inhibition of fatty acid β‐oxidation and increased TG and fatty acids (Chen, Krausz, Shah, Idle, & Gonzalez, 2009; Suciuab et al., 2015). Though these kinds of changes were not detected in the current study, the finding of impaired TCA cycle was consistent with the conclusions by the earlier studies. The impaired TCA cycle was evidenced by decreased [5,6‐^13^C_2_]glucose, the fraction of TG containing [^13^C_2_]glycerol backbones and [^13^C_3_]succinate in the acetaminophen group. Since fatty acid β‐oxidation occurs through the TCA cycle, and the impaired cycle must inhibit the oxidation that may lead to fatty acid and TG increases. The reaction via ME1 is also tightly linked to the TCA cycle as a part of citrate–pyruvate shuttle to produce acetyl‐CoA and NADPH for fatty acid synthesis (Figure 4). Citrate of the cycle in the mitochondrion is exported to the cytosol where it is converted to acetyl‐CoA and oxaloacetate. While the acetyl‐CoA is used for fatty acid synthesis, oxaloacetate is converted to malate and consequently to pyruvate via ME1. Pyruvate after entering the mitochondrion becomes oxaloacetate via carboxylase, or acetyl‐CoA via dehydrogenase, and the condensation of oxaloacetate and acetyl‐CoA yields citrate, completing the shuttle. Thus, it seemed that decreased ME1 protein was caused in part by impaired TCA cycle after acetaminophen treatment.

Glutathione depletion in the liver did not induce either the PPP activity or the regulatory enzyme, G6PDH, protein. The discrepancy between unchanged G6PDH and reduced PPP activity could be the delay of protein synthesis or related with a dynamic turnover of the enzyme expression and degradation. In our recent study with Zucker diabetic fatty rats, both the enzyme and the PPP activity paralleled triglyceride synthesis in the liver (Jin et al., 2018). These findings imply that NADPH from the PPP is important for reductive biosynthesis, but not critical for antioxidant activity in the liver. Like G6PDH, MTHFD1 also remained unaltered in glutathione‐depleted livers. In addition to NADPH regeneration, MTHFD1 and the PPP have another common feature, participating in nucleotide synthesis through one carbon metabolism and by supplying ribose 5‐phosphate, respectively. Together these data suggested that the PPP and MTHFD1 in liver are more critical for anabolism and cell proliferation rather than protection against oxidative stress.

In summary, although the PPP is a ready source of NADPH that is essential for hepatic lipogenesis, its role in protection against oxidative stress was not detected in glutathione‐depleted livers of hamsters. Since the administration of [U‐^13^C_3_]glycerol and analysis of plasma glucose are easily translated to human subjects, these results could be verified in later studies with human patients. Among NADPH‐producing enzymes in the liver, IDH1 was most sensitive to glutathione depletion and it must contribute to recovering redox balance in the cytosol. Glutathione in the cytosol is exported to the mitochondrion, and it remains to be investigated if IDH1 has any significant role for mitochondrial protection against oxidative stress.

## CONFLICT OF INTEREST

The authors declare no competing interests.

## AUTHOR CONTRIBUTIONS

E.S.J. and C.R.M. conceived and designed the research; E.S.J. and M.H.L. performed the experiments and analyzed the data; E.S.J., M.H.L., and C.R.M. interpreted the results of experiments; E.S.J. prepared the figures; E.S.J. drafted the manuscript; E.S.J. and C.R.M. edited and revised the manuscript; E.S.J., M.H.L., and C.R.M. approved the final version of manuscript.
